# 

**Published:** 1907-02

**Authors:** 


					﻿CONTENTS.
ORIGINAL communications—
A Plea for Earlier and More Careful Abdominal Diagnosis By William Perrin Nicolson, M, D.,
Atlanta, Ga._____________________________...______________________________________________7n
Outside and Inside- By C. A Lindorme. M. D.. Atlanta, Ga___________________________________711
A Simple Original Method of Computing Milk Formula. By L. B. Clarke, M. D., Lecturer on Diseases
of Children, Atlanta School of Medicine, Atlanta, Ga..................................   72
Discussion of Dr, Clarke’s Paper. Opened by Dr. C. E. Boynton___________.___________._____..72
Severe Burn from Vaginal Douche. By C. Lambert. M. D., New York City—.....................72
EDITORIAL NOTES AND COMMENTS—
Sulphate of Magnesia...........................................................          .7'
OBITUARIES—
Dr. Charles Davis Hurt..__________________________ ________________________________________.71
Dr. Hunter Pope Cooper..........................................................           T
NEWS AND NOTES_______________________________________________________________________________....71
BOOK REVIEWS____________________________________________________________________________________71
SELECTIONS ANI) ABSTRACTS—
Newer Ideas of the Causes and Treatment of Bright’s Disea=e. By Alfred C. Croftan, Chicago, Ill_7
Purification of Sewage_____________________________________________________________________7
Rapidity in Operating............................................................         7’
Osteopathy in Congress.......................................................             7!
Prevention of Epididymitis_________________________________________________________________7:
When to Use Medicine in Pneumonia_________________________________________________________..71
The Role of the Medical Profession in Combating the Social Evil...................—_________7(
Phthisis and Superstition Among the Moaris_________________________________________________76
A Plea for Early Surgical Interference in Pelvic Infections________________________________76
Treatment of Haemophilia by Injections of Serum..................................        .71
Prolapsus Ani______________________________________________________________________________7f
/ Maxims for the Selection of Climate in Pulmunary, Laryngeal and Bone Tuberculosis____________76
Safety in Marriage_________________________________________________________________________771
And it Wasn’t the Stegomyia Fasciata After All____________________________________../_______77
Railway Accidents_______________________________________________________________________________77
Absorption of Remedies into the Skin________________________________________s__________.________Til
The Omentum and its Functions______________________________________________________________Ti‘
The Toxic Factor in Certain Mental Diseases________________________________________________771
Self-Propelled Vehicles.... ___________________ ___________________________________________77
SURGICAL SUGGESTIONS____________________________________________________________________________77
				

